# On the best proximity point for the proximal contractive and nonexpansive mappings on the starshaped sets

**DOI:** 10.1186/s40064-016-1976-0

**Published:** 2016-03-15

**Authors:** Meifang Guo, Xia Li, Yongfu Su

**Affiliations:** Deprtment of Mathematics and Sciences, Shijiazhuang University of Economics, Shijiazhuang, 050031 People’s Republic of China; Department of Mathematics, Tianjin Polytechnic University, Tianjin, 300387 People’s Republic of China

**Keywords:** Best proximity point, Proximal nonexpansive mapping, Starshaped set, Nonexpansive map

## Abstract

The purpose of this paper is to the best proximity point theorems for the proximal nonexpansive mapping on the starshaped sets by using a clever and simple method. The results improve and extend the recent results of Chen et al. (Fixed Point Theory Appl 2015:19, 2015). It should be noted that, the complex method is used by Jianren Chen et al. can be replaced by the clever and simple method presented in this paper.

## Introduction and preliminaries

The best proximity point problems and best proximity point theorems are basic part of nonlinear analysis and applications. In the recent years, many authors are studying the best proximity point problems. a lot of the best proximity point theorems and relatively results have been obtained in the metric spaces or normed spaces (see Fan 1969; Reich 1978; Prolla 1983; Sehgal and Singh 1988, 1989; Vetrivel et al. 1992; Basha 2000, 2011a, b; Kirk et al. 2003; Veeramani et al. 2005; Eldred and Veeramani 2006; Gabeleh 2013a, b, 2014; Sankar Raj 2011; Abkar and Gabeleh 2013a, b; Kosuru and Veeramani 2011; Lovaglia 1955; Opial 1967; Al-Thagafi and Shahzad 2009; Zhang et al. 2013; Chen et al. 2015; Hussain and Hezarjaribi 2016; Shayanpour et al. 2016; Yongfu and Yao 2015; AlNemer et al. 2016; Samet 2015; Yongfu et al. 2015; Kiran et al. 2015; Binayak 2015; Kong et al. 2015; Yongfu and Zhang 2014; Sun et al. 2014).

Let $T{:}\,A\rightarrow B$, where *A*, *B* are two nonempty subsets of a metric space (*X*, *d*). Note that if $A \cap B = \emptyset$, the equation $Tx = x$ might have no solution. Under this circumstance it is meaningful to find a point $x \in A$ such that *d*(*x*, *Tx*) is minimum. Essentially, if $d(x, Tx) = dist(A,B) = \inf \{d(x, y) {:} \,x \in A, y \in B\}$, *d*(*x*, *Tx*) is the global minimum value *dist*(*A*, *B*) and hence *x* is an approximate solution of the equation $Tx = x$ with the least possible error. Such a solution is known as a best proximity point of the mapping *T*. A point $x \in A$ is called the best proximity point of *T* if

$$\begin{aligned} d(x, Tx) = dist(A,B) = \inf \{ d(x, y) : x \in A, y \in B\}. \end{aligned}$$

It is easy to see that if $A \cap B \ne \emptyset$ , the best proximity point just is the fixed point of *T*. We can find an early classical work in Fan (1969), and afterward, there have been many interesting results such as in Reich (1978), Prolla (1983), Sehgal and Singh (1988, 1989), Vetrivel et al. (1992), Basha (2000, 2011a, b), Kirk et al. (2003), Veeramani et al. (2005) and Eldred and Veeramani (2006), and many others ( for example Gabeleh 2013a, b, 2014; Sankar Raj 2011; Abkar and Gabeleh 2013a, b; Kosuru and Veeramani 2011; Lovaglia 1955; Opial 1967; Al-Thagafi and Shahzad 2009; Zhang et al. 2013; Chen et al. 2015; Hussain and Hezarjaribi 2016; Shayanpour et al. 2016; Yongfu and Yao 2015; AlNemer et al. 2016; Samet 2015; Yongfu et al. 2015; Kiran et al. 2015; Binayak 2015; Kong et al. 2015; Yongfu and Zhang 2014; Sun et al. 2014).

Recently, Gabeleh introduced a new notion which is called the proximal nonexpansive mapping in Gabeleh (2013).

### **Definition 1**

(Gabeleh 2013a) Let (*A*, *B*) be a pair of nonempty subsets of a metric space (*X*, *d*). A mapping $T{:} A\rightarrow B$ is said to be proximal nonexpansive if

$$\begin{aligned} {\left\{ \begin{array}{ll} d(u_1,Tx_1)=dist(A,B)\\ d(u_2,Tx_2)=dist(A,B) \end{array}\right. } \Rightarrow d(u_1,u_2)\le d(x_1,x_2). \end{aligned}$$

for all $$x_1,x_2, u_1,u_2\in A$$.

### **Definition 2**

(Basha 2011a) Let (*A*, *B*) be a pair of nonempty subsets of a metric space (*X*, *d*). A mapping $T{:} \,A\rightarrow B$ is said to be proximal contraction if there exists a constant $0<\alpha <1$ such that

$$\begin{aligned} {\left\{ \begin{array}{ll} d(u_1,Tx_1)=dist(A,B)\\ d(u_2,Tx_2)=dist(A,B) \end{array}\right. } \Rightarrow d(u_1,u_2)\le \alpha d(x_1,x_2). \end{aligned}$$

for all $$x_1,x_2, u_1,u_2\in A$$.

### **Definition 3**

(Kosuru and Veeramani 2011) Let (*A*, *B*) be a pair of nonempty subsets of a metric space (*X*, *d*). The pair (*A*, *B*) is said to be a semi-sharp proximinal pair if for each $x \in A$ (respectively, in *B*) there exists at most one $x^*\in B$ (respectively, in *A*) such that $d(x, x^*) = dist(A,B)$.

The following notions are presented in Chen et al. (2015).

### **Definition 4**

(Chen et al. 2015) A nonempty subset *A* of a linear space *X* is called a *p*-starshaped set if there exists a point $p \in A$ such that $\alpha p + (1-\alpha )x \in A$ , for all $x \in A$, $\alpha \in [0, 1]$, and *p* is called the center of *A*.

Let *A*, *B* be two nonempty subsets of a metric space (*X*, *d*). We denote by $A_0$ and $B_0$ the following sets:

$$\begin{aligned} A_0 & = {} \{x\in A: d(x,y)=d(A,B) \ \ for \ \ some \ \ y\in B \}\\ B_0& = {} \{y\in B: d(x,y)=d(A,B) \ \ for \ \ some \ \ x\in A \} \end{aligned}$$

where $$d(A,B)=\inf \{d(x,y): x\in A \ \ and \ \ y\in B \}.$$

### *Remark 5*

(Chen et al. 2015) It is easy to see that in a normed space $(X, \Vert \cdot \Vert )$, if *A* is a *p*-starshaped set and *B* is a *q*-qstarshaped set and $\Vert p-q\Vert = dist(A,B)$, $A_0$ is a *p*-starshaped set, and $B_0$ is a *q*-starshaped set, respectively. If both of *A* and *B* are closed and $A_0$ is nonempty, $A_0$ is closed.

The purpose of this paper is to the best proximity point theorems for the proximal nonexpansive mapping on a starshaped sets by using a clever and simple method. The results improve and extend the recent results of Chen et al. (2015). It should be noted that, the complex method is used by Jianren Chen et al. can be replaced by the clever and simple method presented in this paper.

## A clever and simple method of proof

In Chen et al. (2015), authors proved the following conclusion, which plays an important role in the proof of their main results. However, the method used in the proof is relatively complicated. In this section, we will give a clever and simple method of proof.

### **Conclusion 1**

(Chen et al. 2015, Lemma 3.1) *Let* (*A*, *B*) *be a pair of nonempty closed subsets of a complete metric space* (*X*, *d*) *and*$A_0$*be closed and nonempty. Assume that*$T{:} A\rightarrow B$*satisfies the following conditions*:

- *T is a proximal contraction*;
- $$T(A_0) \subset B_0$$.

*Then there exists a unique*$x^* \in A$*such that*$d(x^*,Tx^*)=dist (A,B).$

### *Simple proof*

For any $x \in A_0$, since *T* is a proximal contraction, if $d(u_1, Tx)=dist (A,B)$ and $d(u_2, Tx)=dist (A,B)$, then $u_1=u_2$, hence there exists a unique $u \in A_0$ such that $d(u, Tx)=dist (A,B)$, we denote by $u=PTx$ this relation. That is, we define a mapping *P* from $T(A_0)$ into $A_0$. Hence *PT* is a mapping from complete metric subspace $A_0$ into it-self. From (a) we know that,

$$\begin{aligned} d(PTx_1,PTx_2)\le \alpha d(x_1,x_2), \ \ \forall \ x_1,x_2 \in A_0. \end{aligned}$$

By using Banach contraction mapping principle, there exists a unique point $x^* \in A_0$ such that $x^*=PTx^*$, this equivalent to $d(x^*,Tx^*)=dist (A,B)$. The proof is completes. $\square$

The following conclusion is a main result of Jianren Chen et al. The method used in the proof is also complicated. In this section, we also give a very simple method of proof.

### **Conclusion 2**

(Chen et al. 2015, Theorem 3.3) *Let* (*A*, *B*) *be a pair of nonempty, closed subsets of a Banach spaceXsuch thatAis ap*-*starshaped set*, *Bis aq*-*starshaped set, and*$\Vert p-q\Vert = dist(A,B)$. *SupposeAis compact, and* (*A*, *B*) *is a semi*-*sharp proximinal pair. Assume that*$T{:} A \rightarrow B$*satisfies the following conditions*:

- *Tis a proximal nonexpansive*;
- $$T(A_0)\subset B_0$$.

*Then there exists an element*$x^* \in A$*such that*$\Vert x^*-Tx^*\Vert =dist (A,B).$

### *Simple proof*

For any $x \in A_0$, since *T* is a proximal nonexpansive, there exists a unique $u \in A_0$ such that $d(u, Tx)=dist (A,B)$, we denote by $u=PTx$ this relation. Hence *PT* is a mapping from complete metric subspace $A_0$ into it-self. From (a) we know that,

$$\begin{aligned} \Vert PTx_1-PTx_2\Vert \le \Vert x_1-x_2\Vert , \ \ \forall \ x_1,x_2 \in A_0. \end{aligned}$$

Hence *PT* is a nonexpansive mapping from $A_0$ into it-self. We define a mapping $S: A_0\rightarrow A_0$ by

$$\begin{aligned} Sx=\lambda p+(1-\lambda )PTx \end{aligned}$$

where $0<\lambda <1$, then *S* is a contraction. From Remark 5, we know that $A_0$ is closed, by using Banach contraction mapping principle, there exists a unique element $x_\lambda \in A_0$ such that

$$\begin{aligned} x_\lambda =\lambda p+(1-\lambda )PTx_\lambda \end{aligned}$$

for any $0<\lambda <1$. Therefore, there exists a sequence $\{x_n\}$ such that

$$\begin{aligned} x_n=\frac{1}{n} p+\left(1-\frac{1}{n}\right)PTx_n \end{aligned}$$

which implies that

$$\begin{aligned} \Vert x_n-PTx_n\Vert =\left(1-\frac{1}{n}\right)\Vert p-PTx_n\Vert \rightarrow 0. \end{aligned}$$

as $n\rightarrow \infty$, since *A* is compact. On the other hand, there exists a subsequence $\{x_{n_k}\}$ to converge a element $x^* \in A$. Therefore $x^*=PTx^*$, this is equivalent to $d(x^*,Tx^*)=dist (A,B)$. The proof is completes. $\square$

## Further generalized results

By using the same method as in the Conclusion 2, we can get the following further generalized results without assume the (*A*, *B*) is a semi-sharp proximinal pair, and the *A* is compact.

### **Theorem 1**

*Let* (*A*, *B*) *be a pair of nonempty, closed subsets of a Banach spaceXsuch thatAis ap*-*starshaped set*, *Bis aq*-*starshaped set, and*$\Vert p-q\Vert = dist(A,B)$. *SupposeAis weakly compact. Assume that*$T{:} A \rightarrow B$*satisfies the following conditions*:

- *Tis a proximal nonexpansive*;
- $$T(A_0)\subset B_0$$.

*Then there exists an element*$x^* \in A$*such that*$\Vert x^*-Tx^*\Vert =dist (A,B).$

### *Proof*

Since *A* is weak compact, there exists a subsequence $\{x_{n_k}\}$ to converge weakly a element $x^* \in A$. Noting that, the nonexpansive mapping *PE* is demi-closed, then $x^*=PTx^*$, this is equivalent to $d(x^*,Tx^*)=dist (A,B)$. The proof is completes. $\square$

### **Theorem 2**

*LetXbe a uniformly convex Banach space with the Opail’s condition. Let* (*A*, *B*) *be a pair of nonempty, closed subsets ofXsuch thatAis ap*-*starshaped set*, *Bis aq*-*starshaped set, and*$\Vert p-q\Vert = dist(A,B)$. *SupposeAis bounded. Assume that*$T{:}\, A \rightarrow B$*satisfies the following conditions*:

- *Tis a proximal nonexpansive*;
- $$T(A_0)\subset B_0$$.

*Then there exists an element*$x^* \in A$*such that*$\Vert x^*-Tx^*\Vert =dist (A,B).$

### *Proof*

Since *A* is bounded so it is weak compact, there exists a subsequence $\{x_{n_k}\}$ to converge weakly a element $x^* \in A$. Noting that, since *X* satisfies Opail’s condition, the nonexpansive mapping *PE* must be demi-closed (see Lemma 2 in Opial 1967), then $x^*=PTx^*$, this is equivalent to $d(x^*,Tx^*)=dist (A,B)$. The proof is completes. $\square$

Let $g{:} A_0\rightarrow A_0$ be an isometry, we replace by $S=g^{-1}PT$ the $S=PT$, respectively in Theorems 1 and 2, we can get the following results which are further generalized forms to the result of Chen et al. (2015, Theorem 3.4).

### **Theorem 3**

*Let* (*A*, *B*) *be a pair of nonempty, closed subsets of a Banach spaceXsuch thatAis ap*-*starshaped set*, *Bis aq*-*starshaped set, and*$\Vert p-q\Vert = dist(A,B)$. *SupposeAis weakly compact. Assume that*$T{:} \,A \rightarrow B , g: A_0\rightarrow A_0$*satisfy the following conditions*:

- *Tis a proximal nonexpansive*;
- $$T(A_0)\subset B_0$$.
- *gis an isometry*.

*Then there exists an element*$x^* \in A$*such that*$\Vert gx^*-Tx^*\Vert =dist (A,B).$

### **Theorem 4**

*LetXbe a uniformly convex Banach space with the Opail’s condition. Let* (*A*, *B*) *be a pair of nonempty, closed subsets ofXsuch thatAis ap*-*starshaped set*, *Bis aq*-*starshaped set, and*$\Vert p-q\Vert = dist(A,B)$. *SupposeAis bounded. Assume that*$T {:} A \rightarrow B , g{:} A_0\rightarrow A_0$*satisfy the following conditions*:

- *Tis a proximal nonexpansive*;
- $$T(A_0)\subset B_0$$.
- *g* is an isometry.

*Then there exists an element*$x^* \in A$*such that*$\Vert gx^*-Tx^*\Vert =dist (A,B).$

### **Definition 6**

(Chen et al. 2015) Let (*A*, *B*) be a pair of nonempty subsets of a metric space (*X*, *d*). The pair (*A*, *B*) is said to have the weak *P*-property if

$$\begin{aligned} {\left\{ \begin{array}{ll} d(x_1,y_1)=dist(A,B)\\ d(x_2,y_2)=dist(A,B) \end{array}\right. } \Rightarrow d(x_1,x_2)\le d(y_1,y_2). \end{aligned}$$

for all $$x_1,x_2 \in A_0, y_1,y_2\in B_0$$.

By using the well-known Schauder fixed point theorem, we can improve the result of (Theorem 4.1, Chen et al. 2015) to the following generalized result.

### **Theorem 5**

*Let* (*A*, *B*) *be a pair of nonempty, closed, and convex subsets of a Banach spaceXsuch thatAis compact. Suppose that*$A_0$*is nonempty and* (*A*, *B*) *has the weakP*-*property. Let*$T{:}\,A \rightarrow B$*be a nonexpansive non-self-mapping such that*$T(A_0) \subset B_0$. *ThenThas at least one best proximity point inA*.

### *Proof*

In this case, the mapping *PT* defined in Conclusion 2 is also a nonexpansive mapping from $A_0$ into it-self. Since *A* is compact and convex, by using Schauder fixed point theorem, there exists a element $x^* \in A$ such that $x^*=PTx^*$, this is equivalent to $\Vert x^*-Tx^*\Vert =dist (A,B).$ This completes the proof. $\square$
